# Editorial: Rising stars in psychopathology research

**DOI:** 10.3389/fpsyg.2023.1264864

**Published:** 2023-08-25

**Authors:** Bohua Hu, Qinghua He, Xavier Noel, Antoine Bechara, Éric Laurent

**Affiliations:** ^1^Faculty of Psychology, Southwest University, Chongqing, China; ^2^Ministry of Education Key Laboratory of Cognition and Personality, Southwest University, Chongqing, China; ^3^Laboratoire de Psychologie Médicale et d'Addictologie, Faculty of Medicine, Université Libre de Bruxelles (ULB), Brussels, Belgium; ^4^Department of Psychology, University of Southern California, Los Angeles, CA, United States; ^5^Department of Psychology, Université de Franche-Comté, Besançon, France

**Keywords:** rising stars, psychopathology, mental health, mental disorders, editorial

Theories and research methods in psychopathology depend on the specific social, cultural and scientific context (Telles-Correia and Sampaio, 2016). There have been multiple paradigm shifts in the development history of psychopathology, which has spanned more than a hundred years. From psychoanalysis and phenomenology (existentialism) to cognitive science, biological psychotherapy, and molecular neuroscience (Kendler, 2008), the theories of psychopathology have been continuously updated and the research techniques of psychopathology have become more extensive. Technological and theoretical innovations enable research to have a deeper and more comprehensive understanding of the identification, potential mechanisms, and treatment plans of various mental illnesses and mental symptoms. Therefore, this Research Topic has assembled a number of high-quality research that have made new progress in research techniques and theoretical construction, including one review (Ke et al.) and four original research (Dust; Kornacka et al.; Li et al.; Otte et al.), demonstrating new directions and trends in psychopathology research.

## Review

Ke et al. conducted a systematic review of research papers on Developmental Coordination Disorders (DCD) in past decades. They noted that since the unification of the DCD definition in 2013, the number of published related research papers has been steadily increasing, particularly in developing countries. These papers come from different disciplines, mainly including cognitive neuroscience, psychology, clinical medicine and physical education, focusing on different aspects of DCD research. Over the last 3 years, research in this field has increased interest in neurodevelopmental mechanisms, perinatal factors, proteomics and paid more attention to the combination of multidisciplinary and multimodal data. As a result, some large cohort study databases are increasingly incorporating such comprehensive data. Based on a large, publicly accessible database, researchers can use the massive comprehensive data to study subtype classification and cognitive neural mechanisms of DCD, which is crucial for guiding early screening, diagnosis, and intervention in children with DCD.

## Original research

In a cross-sectional study, Otte et al. explored the effects of the COVID-19 pandemic on obsessive-compulsive symptoms (OCSs) in a large sample of the general population. By analyzing participants' scores on two Obsessive-Compulsive Inventory-Revised (OCI-R) questionnaires (one for assessing OCSs during the pandemic and the other for retrospectively assessing OCSs before the pandemic), they found that more participants reported clinically relevant OCSs during the pandemic compared to pre-pandemic, and the severity of participants' OCSs significantly increased during the pandemic, particularly in the washing dimension. Furthermore, a weak positive association was observed between the increased severity of OCSs and self-reported stress and anxiety. This study is the first to investigate the possible impact of the COVID-19 pandemic on OCSs and clinically relevant changes in OCSs during the pandemic in a large sample of the general population, which is important for identifying at-risk groups and developing relevant countermeasures during the COVID-19 pandemic.

Dust's study investigated whether the Community Resiliency Model (CRM) or Mental and Emotional Self-Management (MESM) intervention could boost physiological resilience to prevent posttraumatic stress disorder (PTSD) and what are the physiological mechanisms behind their respective effects. In this study, A non-traumatized sample was split into three groups: CRM, MESM and Control, all of which were required to participate in a pretest, an intervention/control and a posttest, both pretest and posttest required participants to watch some stimulating videos and measured their cardiac vagal tone before and after watching. The results revealed that only in the posttest of the MESM group, the cardiac vagal tone after watching the video was higher than before, which may imply a better stress processing ability. This research is based on potentially trainable physiological processes and provides a foundation for future studies on the physiological resilience of traumatic stress.

Using the resting-state EEG microstate analysis, Li et al. explored the neurophysiological mechanism of adolescent smoking addiction. They compared the duration, occurrence, coverage and transition probabilities of microstates A, B, C, and D between young male smokers and non-smoking controls. The results showed that young male smokers had significantly less occurrence of microstate C and significantly longer duration of microstate D than non-smokers, indicating changes in specific brain functions in these smokers. In addition, the duration and coverage of microstate D were negatively correlated to nicotine dependence, suggesting that the altered features of microstate D may be a possible biomarker for predicting nicotine dependence in young male smokers. As a whole-brain imaging approach, EEG microstate analysis can assess the function of large-scale brain networks with high temporal resolution. The discovery of abnormal EEG microstates in adolescent smokers provides a new perspective for future studies of the neural mechanisms of smoking addiction.

Kornacka et al. conducted an experience sampling study to test two hypotheses used to explain the occurrence of maladaptive task-unrelated thoughts (TUT). The context regulation theory suggests that task difficulty and self-control resources could affect the intensity of TUT. More specifically, individuals will have a lower TUT level in difficult and cognitively demanding tasks compared to simple tasks, but a failure of self-control will result in high and maladaptive TUT occurrence during difficult tasks. The avoidant alternative hypothesis complemented the previous theory, it suggests that the negative valence of tasks will also lead to the maladaptive TUT occurrence during difficult tasks. Moreover, the interaction effect of task valence and task difficulty on TUT intensity will be moderated by one's emotion regulation strategy. Both hypotheses were verified in this study. This is the first study to empirically confirm the avoidant alternative hypothesis, providing preliminary directions for studying the mechanisms and solutions of maladaptive TUT, as well as the evaluation and diagnosis of related mental disorders.

In summary, this Research Topic has assembled a review and four original research that have made new advances in research methodology and theoretical construction in psychopathology. We hope that these achievement will help researchers to have a more comprehensive understanding and exploration of mental illnesses and mental symptoms.

## Author contributions

BH: Writing—original draft, Writing—review and editing. QH: Funding acquisition, Supervision, Writing—original draft, Writing—review and editing. XN: Writing—review and editing. AB: Writing—review and editing. ÉL: Writing—review and editing.

## References

[B1] Kendler K. S. (2008). “Introduction: why does psychiary need philosophy?” in Philosophical Issues in Psychiary, eds K. S. Kendler and J. Parnas (Baltimmore, MD: The Johns Hopkins University Press), 1–18.

[B2] Telles-Correia D. Sampaio D. (2016). Historical roots of psychopathology. Front. Psychol. 7, 905. 10.3389/fpsyg.2016.00905 27378995 PMC4905962

